# Beyond the Hazard Ratio: Rethinking Survival Reporting in Cardio-Oncology With Restricted Mean Survival Time

**DOI:** 10.1016/j.jaccao.2025.11.003

**Published:** 2026-02-17

**Authors:** Jean Henri Maselli-Schoueri, Samuel David Saibil, Victor Gabriel da Silva Negrini, Ary Serpa Neto, Marcus Butler

**Affiliations:** a Princess Margaret Cancer Centre, Department of Medicine, University of Toronto, Toronto, Ontario, Canada; b Melanoma & Skin Oncology, Princess Margaret Cancer Centre, Division of Medical Oncology and Hematology, Department of Medicine, University of Toronto, Toronto, Ontario, Canada; c Instituto de Cardiologia Dante Pazzanese, São Paulo, Brazil; d Department of Intensive Care, Austin Hospital, Melbourne, Australia

**Keywords:** cancer survivorship, immunotherapy, melanoma, myocarditis

**Figure:**
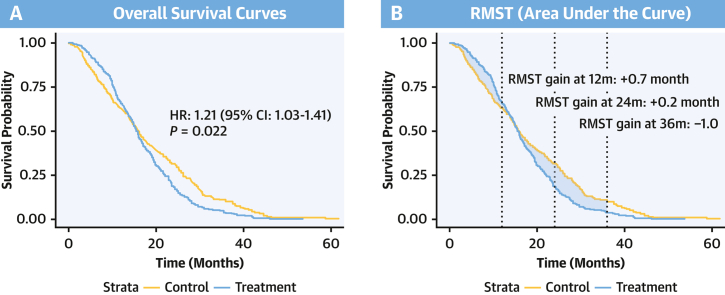

Immune checkpoint inhibitors (ICIs) have revolutionized oncology. As seen in clinical trials over the past decade, ICI treatment has shifted the prognosis, providing survival benefits far beyond the median while creating a survival “tail” that challenges traditional interpretation. Standard methods, such as the log-rank test and Cox proportional hazards model, presume a constant effect over time, an assumption often violated in trials showing early toxicity and late benefit.^1^ In cardio-oncology, this is especially relevant, as acute myocarditis may influence early mortality, while accelerated atherosclerosis may be clinically evident later.^2^^,^^3^ Furthermore, the mere rate of treatment discontinuation due to immune-related toxicity can modify survival patterns, emphasizing the importance of competing-risks and time-dependent analyses.

When proportional hazards do not hold, median survival and HRs can obscure true survival benefit and patient-centered outcomes, as reflected in recent assessments highlighting inflated gains in overall survival and limited improvements in quality of life.^4^^,^^5^ In this scenario, the restricted mean survival time (RMST) provides a complementary framework for interpreting such complex outcomes. Put simply, RMST can be intuitively understood as the average survival time, or average event-free time, up to a prespecified time horizon, that is, the area under the Kaplan-Meier curve up to a selected truncation point (τ).^6^ This point is typically defined by the last event observed in both treatment arms (Figure 1). Methodological literature supports the RMST as a complement to the log-rank test that does not rely on the proportional hazards assumption, yet most trials rarely report it systematically.^7^

**Figure 1:**
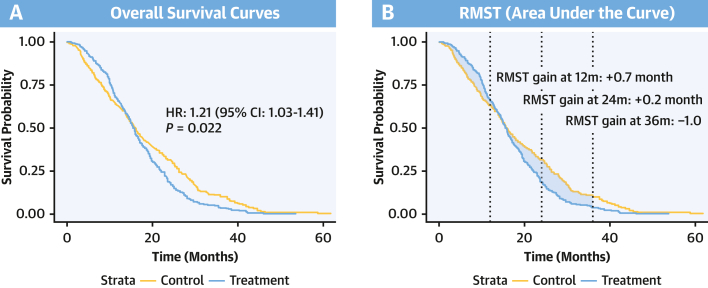
Schematic Comparison of HR and RMST Estimates Under Nonproportional Hazards With Crossing Curves Restricted mean survival time (RMST) differences reflect time-dependent treatment effects. An early gain (at 12 months) may diminish at 24 months if survival curves cross over or overlap, commonly seen when control arm patients catch up or when early toxicity affects treated patients. A renewed benefit at 36 months often signals a durable response, as seen with immunotherapy compared with chemotherapy. This dynamic insight is often lost in a single HR estimate (A), and this time-varying pattern is more transparently summarized by RMST (B).

Most important, RMST answers a clinically meaningful question: “How much additional time does this treatment provide?” We recommend that trials with planned long-term follow-up or time-varying effects routinely report the RMST difference alongside traditional log-rank and HR estimates. This guidance does not replace the log-rank test but adds a measure better aligned with patient-centered clinical decisions, a basis that encourages transparent communication between physicians and patients. Additionally, it provides an absolute measure of survival gain that can be directly integrated into health economic models, facilitating more meaningful cost-effectiveness analyses.^8^

It must be noted, however, that the RMST should be used as a complementary tool rather than a panacea. Despite its multiple advantages, when used in isolation it also presents limitations.^7^ First, it depends on the truncation point, which may vary across trials, thus creating a new challenge for cross-study comparisons. Moreover, when survival curves cross, a single RMST metric may not be enough to distinguish short- and long-term treatment effects, as early and late hazards may differ substantially across follow-up periods^7^ (Figure 1).

It is time to recognize that relying solely on log-rank tests risks misleading clinical judgment before data maturation and overlooks meaningful patient benefit, ultimately compromising shared decision-making and the integrity of any cost-effectiveness assessment. We encourage cardio-oncology researchers, regulators, and payers to consistently include RMST reporting alongside traditional log-rank and HR estimates to better represent true patient-centered benefits and risks: it is a straightforward metric that helps communicate realistic survival expectations while accounting for the impact of life-threatening toxicities ranging from acute myocarditis to chronic atherosclerosis, leading to worse survival. Importantly, RMST inherently reflects cumulative and long-term effects of therapy, which is consistent with recent frameworks proposed to assess enduring outcomes beyond the proportional hazards assumption.^9^

Clinical guidelines and health technology assessments should account for time-varying effects using methods that match biological reality. We acknowledge that regulatory approval and reimbursement decisions are often made before long-term overall survival data are available and that follow-up is frequently discontinued after 5 years, even when treatment benefits may extend for decades. In such scenarios, RMST provides a statistically valid and clinically interpretable alternative to median or HR-based estimates. Although regulatory agencies have acknowledged delayed curve separation and nonproportional hazards as major analytical challenges in the era of immuno-oncology, RMST remains underused, and proportional hazards are still often overlooked in many health technology assessment submissions, including those from Australia, Europe, and Canada.^10^ By combining traditional and modern survival measures, the transparency of trial evidence can be enhanced, be made more clinically meaningful, and better address the real needs and expectations of patients and payers alike. Our analysis of trial results should serve to inform, and RMST represents a definitive step toward clarity in our everyday practice.

## Funding Support and Author Disclosures

The authors have reported that they have no relationships relevant to the contents of this paper to disclose.
